# Characteristics of Hard Tick Relapsing Fever Caused by *Borrelia miyamotoi*, United States, 2013–2019

**DOI:** 10.3201/eid2909.221912

**Published:** 2023-09

**Authors:** David W. McCormick, Catherine M. Brown, Jenna Bjork, Kim Cervantes, Brenda Esponda-Morrison, Jason Garrett, Natalie Kwit, Abigail Mathewson, Charles McGinnis, Marco Notarangelo, Rebecca Osborn, Elizabeth Schiffman, Haris Sohail, Amy M. Schwartz, Alison F. Hinckley, Kiersten J. Kugeler

**Affiliations:** Centers for Disease Control and Prevention, Fort Collins, Colorado, USA (D.W. McCormick, A.M. Schwartz, A.F. Hinckley, K.J. Kugeler);; Massachusetts Department of Public Health, Boston, Massachusetts, USA (C.M. Brown);; Minnesota Department of Health, Minneapolis, Minnesota, USA (J. Bjork, E. Schiffman);; New Jersey Department of Health, Trenton, New Jersey, USA (K. Cervantes);; Connecticut Department of Public Health, Hartford, Connecticut, USA (B. Esponda-Morrison);; Rhode Island Department of Health, Providence, Rhode Island, USA (J. Garrett, C. McGinnis);; Vermont Department of Health, Burlington, Vermont, USA (N. Kwit);; New Hampshire Department of Health and Human Services, Concord, New Hampshire, USA (A. Mathewson, M. Notarangelo);; Wisconsin Department of Health Services, Madison, Wisconsin, USA (R. Osborn);; Maine Center for Disease Control and Prevention, Augusta, Maine, USA (H. Sohail)

**Keywords:** hard tick relapsing fever, Borrelia miyamotoi, bacteria, tick-borne infections, Ixodes scapularis, ticks, emerging infectious diseases, vector-borne infections, characteristics, zoonoses, United States

## Abstract

*Borrelia miyamotoi*, transmitted by *Ixodes* spp. ticks, was recognized as an agent of hard tick relapsing fever in the United States in 2013. Nine state health departments in the Northeast and Midwest have conducted public health surveillance for this emerging condition by using a shared, working surveillance case definition. During 2013–2019, a total of 300 cases were identified through surveillance; 166 (55%) were classified as confirmed and 134 (45%) as possible. Median age of case-patients was 52 years (range 1–86 years); 52% were male. Most cases (70%) occurred during June–September, with a peak in August. Fever and headache were common symptoms; 28% of case-patients reported recurring fevers, 55% had arthralgia, and 16% had a rash. Thirteen percent of patients were hospitalized, and no deaths were reported. Ongoing surveillance will improve understanding of the incidence and clinical severity of this emerging disease.

Tickborne diseases are an increasing public health problem, accounting for ≈75% of reported vectorborne illnesses in the United States (*1**‒**3*). Continued discovery of new tickborne pathogens in recent years suggests they remain an underrecognized cause of human illness (*4**–**6*). *Borrelia miyamotoi* is a gram-negative spirochete transmitted by *Ixodes* spp. ticks (*7**–**9*) that was initially identified in ticks in Japan during 1995 (*7*). It was recognized as a cause of human illness in Russia during 2011 (*10*) and in the United States during 2013 (*11*). Human infection has since been detected throughout the Holarctic region (*10**,**12**–**17*).

Phylogenetically, *B. miyamotoi* is a relapsing fever group *Borrelia* (*18*). Diseases caused by this diverse group of spirochetes are differentiated by their vector, such as louseborne relapsing fever, transmitted by body lice, and tickborne relapsing fever or soft tick relapsing fever, transmitted by soft-bodied (argasid) ticks in several areas, including the western United States (*19*). *B. miyamotoi* is an agent of hard tick relapsing fever (HTRF), although resulting illness has also been referred to as *B. miyamotoi* disease. In the United States, *B. miyamotoi* is transmitted by *I. scapularis* ticks in the Northeast and Midwest (*20*,*21*) and by *I. pacificus* ticks on the Pacific Coast (*22*). Those tick species also transmit the causative agents of Lyme disease (*23*), anaplasmosis (*24*), babesiosis (*25*), Powassan virus disease (*26*), and a form of ehrlichiosis (*27*). Data from tick testing indicate that the geographic range of *B. miyamotoi* is similar to that of those pathogens (*28*,*29*).

The incidence of HTRF caused by *B. miyamotoi* and its public health role are largely unknown. In the United States, prevalence of *B. miyamotoi* in *Ixodes* spp. ticks is relatively low, but consistent across geographic regions at ≈2% (*3*,*29*). A seroprevalence evaluation conducted in several states in the northeastern United States in 2018 suggested that 2.8% of persons might have evidence of previous infection, compared with 11% of persons who had evidence of previous Lyme disease (*30*). Data from previous case series suggest that HTRF caused by *B. miyamotoi* most often manifests as a nonspecific febrile illness. Among identified cases, fever, myalgia, arthralgia, and headache are common, but recurring fevers similar to those documented in patients who have soft tick relapsing fever are relatively uncommon (4%–11% of total) (*10*,*17*). Immunocompromised persons who have HTRF might have more severe symptoms, including meningoencephalitis (*11*,*16*,*31*).

Specific laboratory diagnosis of *B. miyamotoi* infection is achieved through PCR detection of *B. miyamotoi* DNA (*10*,*17*,*32*). Serologic reactivity to surface proteins, especially glycerophosphodiester phosphodiesterase (GlpQ), is also used, but reactivity is not specific to *B. miyamotoi* infection or HTRF (*33*,*34*). GlpQ is found in all relapsing fever group borreliae but not in the *B. burgdorferi* sensu lato species that cause Lyme disease (*34*). However, GlpQ cannot distinguish between *B. miyamotoi* infection and infections caused by other relapsing fever group *Borrelia* spp., including agents of soft tick relapsing fever. In addition, related GlpQ proteins are found in common bacterial pathogens, such as *Haemophilus influenzae* and *Escherichia coli,* further reducing specificity of those serologic assays (*34*).

After initial cases of HTRF were identified in the United States, several states that had a high incidence of Lyme disease and other *Ixodes*-transmitted illnesses initiated public health surveillance to clarify the epidemiology of this novel tickborne condition. We summarize available information on HTRF as ascertained through public health surveillance efforts in the United States beginning in 2013.

## Methods

### Case Definition

The Centers for Disease Control and Prevention (CDC) and state health departments in areas that had a high incidence of Lyme disease jointly created an informal working surveillance case definition to identify and classify potential cases of HTRF caused by *B. miyamotoi* in a standardized manner. Clinical manifestations considered compatible with HTRF were broadly defined as acute onset of fever or chills with >1 of the following additional signs or symptoms: headache, sweats/chills, myalgia, arthralgia, malaise/fatigue, rash, abdominal cramps, nausea, vomiting, diarrhea, dizziness, confusion/altered mental status, photophobia, leukopenia, thrombocytopenia, or increased aminotransferase levels.

For purposes of surveillance data as summarized, we defined a confirmed case of HTRF caused by *B. miyamotoi* as compatible clinical manifestations with >1 of the following: isolation of *B. miyamotoi* from a clinical specimen; detection of *B. miyamotoi* DNA in a clinical specimen by using nucleic acid amplification techniques (NAAT) such as PCR; or evidence of seroconversion between acute phase and convalescent phase serum samples, including but not limited to a >4-fold change in serum antibody titer to *B. miyamotoi* between paired specimens. We defined a possible case as compatible clinical manifestations with >1 of the following: direct observation of spirochetes consistent with *B. miyamotoi* on a peripheral blood smear or detectable IgM or IgG to *B. miyamotoi* from a serum specimen.

We characterized cases as possible rather than probable to reflect the uncertainty of the spectrum of clinical manifestations of HTRF and the specificity of a single positive serologic titer. We excluded positive laboratory test results for which no clinical information was obtained or for which there was no associated clinical illness. Data on specific test manufacturers or antigenic targets used in serologic assays were not available to ascertain level of specificity for *B. miyamotoi* versus other relapsing fever *Borrelia* spp.

### Public Health Investigation

Commercial or clinical laboratories reported positive laboratory results for *B. miyamotoi* in accordance with local regulations in states in which *B. miyamotoi* infection/HTRF was a reportable condition. Public health personnel conducted case investigations according to local practices to ascertain demographic, clinical, and exposure information to the extent possible through patient or provider interviews or medical chart reviews.

### Analytic Methods

We classified symptoms as present or absent. We categorized age as <18 years, 18–64 years, and >65 years. We compared categorical and binary variables by using χ^2^ or Fisher exact tests and continuous variables by using the Wilcoxon rank-sum test. We performed all statistical analyses by using SAS software (SAS Institute). This study was deemed to be a nonresearch activity by CDC under provision of public health surveillance.

## Results

**Figure 1:**
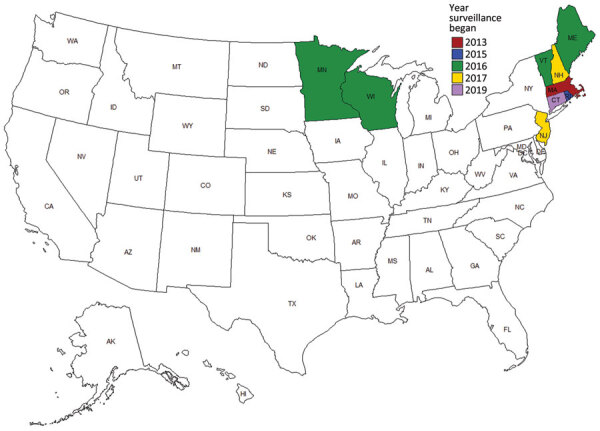
US states that conducted surveillance for hard tick relapsing fever caused by *Borrelia miyamotoi* during 2013–2019 and year in which surveillance began.

**Figure 2:**
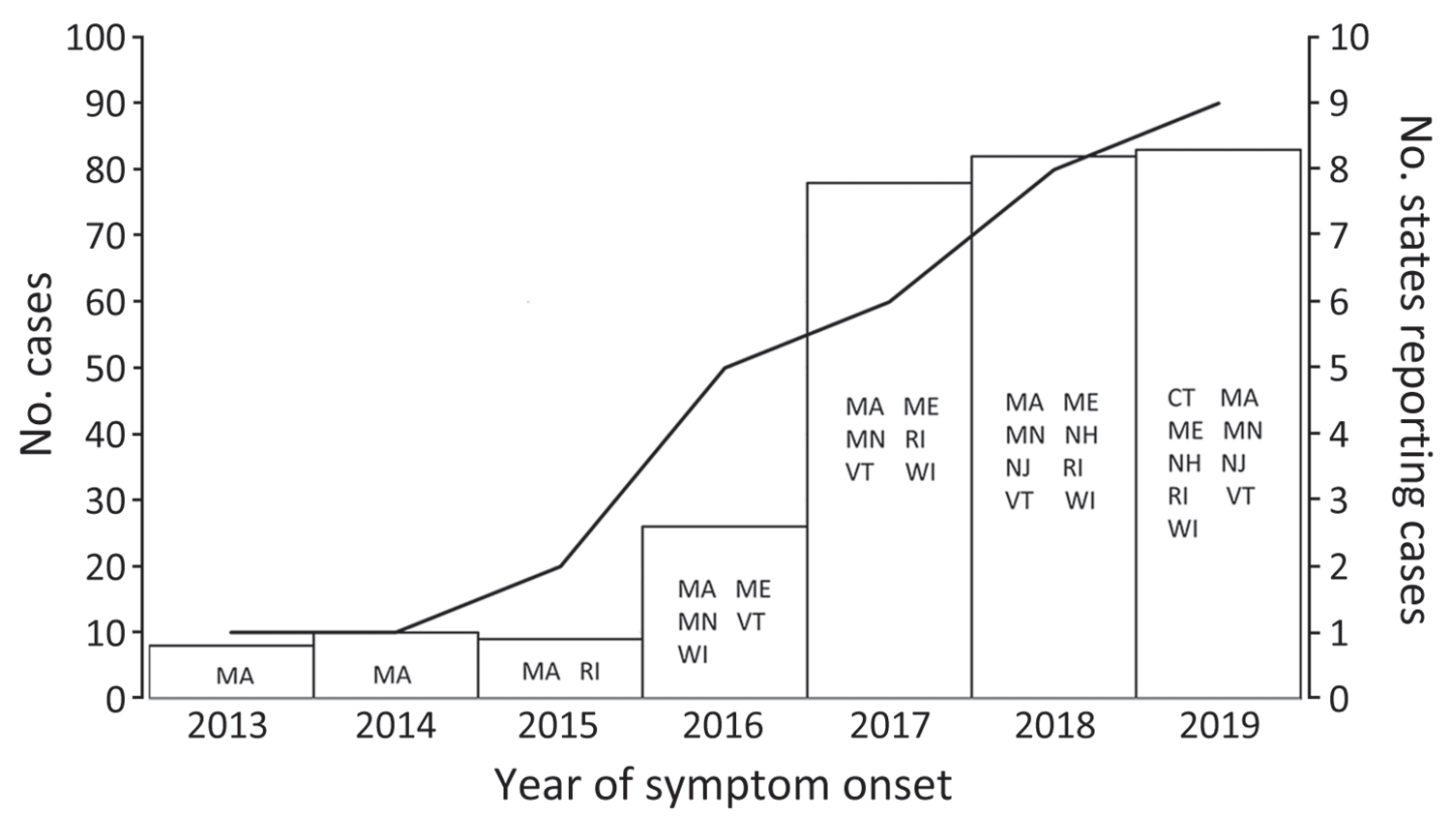
Number of annual cases of hard tick relapsing fever (vertical bars) and number of states reporting cases of hard tick relapsing fever caused by *Borrelia miyamotoi* (line), United States, 2013–2019. The left y-axis corresponds to the vertical bars, and the right y-axis corresponds to the line; scales for the y-axes differ substantially to underscore patterns but do not permit direct comparisons. States reporting cases in that year are shown. CT, Connecticut; MA, Massachusetts; ME, Maine; MN, Minnesota; NH, New Hampshire; NJ, New Jersey; RI, Rhode Island; VT, Vermont; WI, Wisconsin.

A total of 300 HTRF cases caused by *B. miyamotoi* were identified during 2013–2019 by the 9 state health departments in the Northeast and upper Midwest United States that conducted public health surveillance for this condition (Connecticut, Maine, Massachusetts, Minnesota, New Hampshire, New Jersey, Rhode Island, Vermont, and Wisconsin). The number of states in which HTRF was reportable increased from 1 in 2013 to 9 by 2019 (Figure 1). The number of cases identified annually concomitantly increased; more cases were identified during 2017–2019 (median 82, range 78–83 cases/y) than during 2013–2015 (median 9, range 8–10 cases/y) (Figure 2).

**Figure 3:**
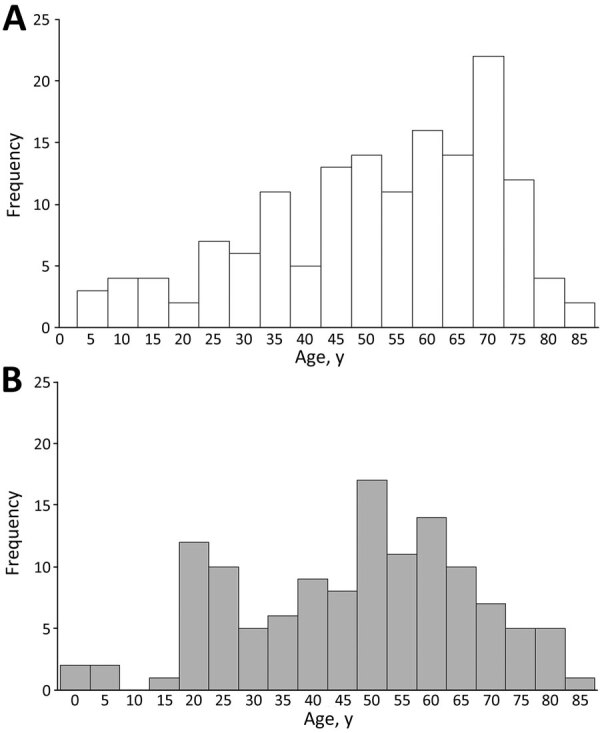
Patient age distribution for confirmed (A) and possible (B) cases of hard tick relapsing fever caused by *Borrelia miyamotoi* identified by using public health surveillance, United States, 2013–2019.

**Figure 4:**
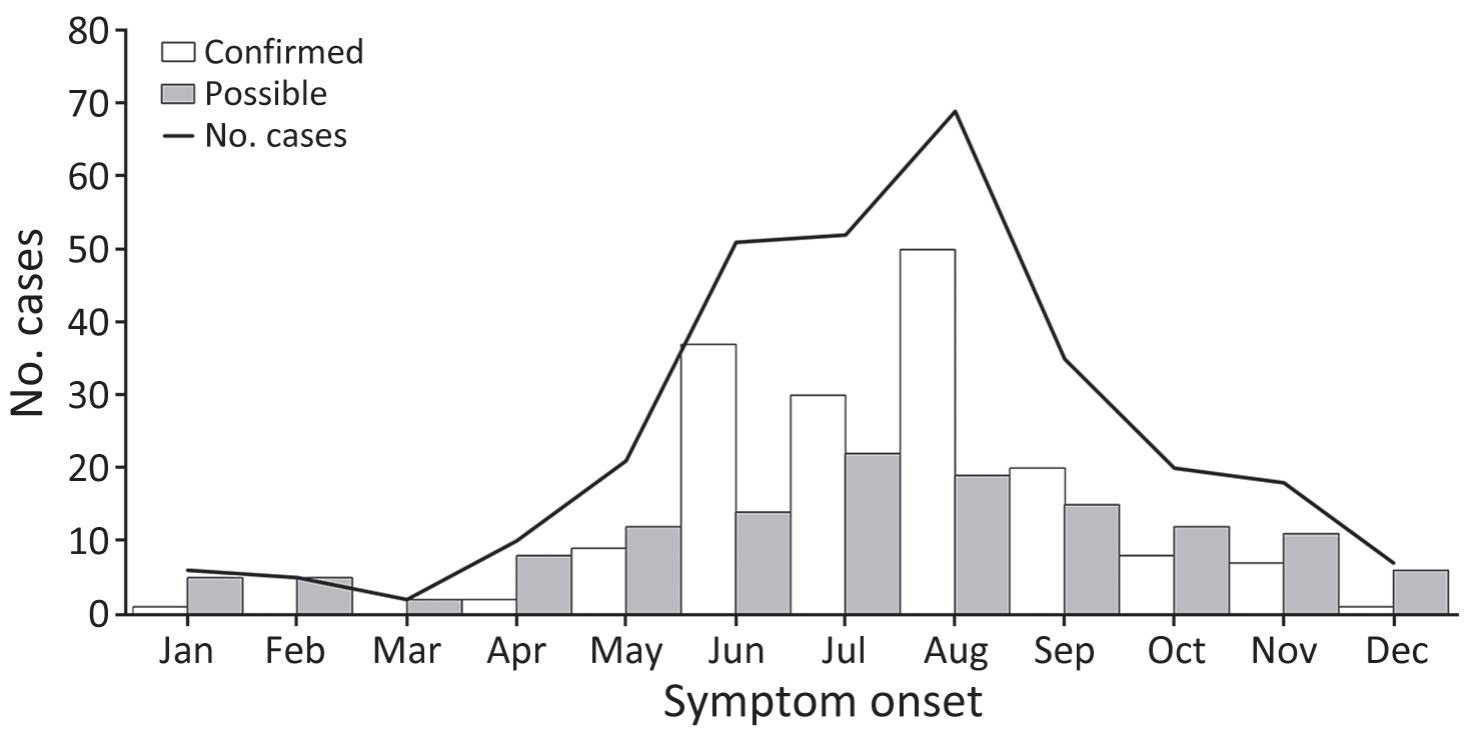
Reported cases of hard tick relapsing fever caused by *Borrelia miyamotoi* by month of symptom onset, United States, 2013–2019. Solid line indicates total number of reported cases each month, and bars indicate number of confirmed (white) and possible (gray) cases each month.

Of the 300 identified cases, 166 (55%) were classified as confirmed and 134 (45%) as possible. Of the 300 cases, 157 (52%) were in male and 143 (48%) among female patients; median age was 52 (range 1–86) years. Almost all cases were among non-Hispanic White persons (107/110, 97%). Median age of persons who had confirmed illness was older than persons who had possible illness (median age 56 [range 4–86] years vs. median 50 [1–86] years; p = 0.03) (Table 1). A higher proportion of confirmed versus possible cases occurred among persons <18 years of age (9% vs. 4%) and among persons >65 years of age (34% vs. 21%; p = 0.004) (Figure 3). Among confirmed cases, 56% of patients were male and 44% female; among possible cases, 49% of patients were male and 51% female (p = 0.23). Most case-patients had symptom onset during June–September, with a peak in August (Figure 4). Compared with confirmed HTRF illness, possible illness had a less pronounced seasonal pattern.

**Table 1:** Demographic characteristics for confirmed and possible cases of hard tick relapsing fever caused by *Borrelia miyamotoi* identified by public health surveillance, United States, 2013–2019*

Characteristic	Confirmed, n = 166	Possible, n = 134	p value
Median age, y (range)†	56 (4–86)	50 (1–86)	0.03‡
Categorical age			
<18	15/166 (9)	5/134 (4)	
18–65	95/166 (57)	101/134 (75)	0.004
>65	56/166 (34)	28/134 (21)	
Sex			
M	92/166 (56)	65/134 (49)	0.23
F	74/166 (44)	69/134 (51)	
Year of illness onset§			
2013	8/165 (5)	0/132 (0)	
2014	9/165 (5)	1/132 (1)	
2015	1/165 (1)	8/132 (6)	
2016	16/165 (10)	10/132 (8)	<0.0001¶
2017	56/165 (34)	22/132 (17)	
2018	38/165 (23)	44/132 (34)	
2019	37/165 (22)	46/132 (35)	

*Values are no. positive/no. tested (%) unless indicated otherwise. †Missing information for 25 persons. ‡By Wilcoxon rank-sum test. §Missing information for 4 persons. ¶By Fisher exact test.

The median duration of time from symptom onset to seeking medical attention was 5 (range 0–311) days for the 69 persons for whom this information was available (Table 2). Persons who had possible illness had a longer duration from symptom onset to medical attention (median 9, interquartile range [IQR] 3–29 days) than persons who had confirmed cases (median 3, IQR 2–7 days; p = 0.03). Overall, the most common symptoms were fever (89%), fatigue (75%), headache (72%), and chills (68%). Among 64 patients who had fever for whom a temperature was available, the median recorded temperature was 102.5°F (range 99.7°F –105.7°F). A total of 28% reported recurring fevers of some kind, 55% had arthralgia, and 16% had a rash. A description of the rash was available for 18 patients; the rash was noted to be generalized for 3 patients (1 confirmed and 2 possible cases) and focal for 15 patients (9 confirmed and 6 possible cases). An erythema migrans‒like rash was reported for 1 possible HTRF case. Thrombocytopenia (51/105, 49%), increased levels of aspartate and alanine aminotransferases (40/96, 42%), and leukopenia (39/105, 37%) were common laboratory abnormalities.

**Table 2:** Clinical and laboratory findings for persons who have confirmed and possible hard tick relapsing fever caused by *Borrelia miyamotoi* identified by public health surveillance, United States, 2013–2019*

Characteristic	Confirmed, n = 165	Possible, n = 133	p value
Hospitalized	20 (12)	19 (14)	0.61
Median duration of illness, d† (IQR)	3 (2–7)	9 (3–29)	0.03
Required symptoms			
Fever	157 (95)	108 (81)	<0.0001
Chills	115 (70)	87 (65)	0.27
Supporting signs and symptoms			
Headache	118 (72)	96 (72)	0.85
Myalgia	104 (63)	94 (71)	0.20
Arthralgia	79 (48)	84 (63)	0.02
Malaise/fatigue	125 (76)	99 (74)	0.55
Rash	21 (13)	28 (21)	0.06
Abdominal pain	16 (10)	27 (20)	0.01
Nausea	55 (33)	36 (27)	0.26
Vomiting	23 (14)	15 (11)	0.44
Diarrhea	8 (5)	17 (13)	0.01
Dizziness	26 (16)	33 (25)	0.06
Confusion	7 (4)	24 (18)	<0.0001
Photophobia	8 (5)	11 (8)	0.29
Leukopenia‡	31 (46)	8 (22)	0.02
Thrombocytopenia§	40 (58)	9 (25)	0.001
Increased levels of aminotransferases¶	27 (45)	13 (36)	0.41
Other symptoms			
Recurring fevers	37 (22)	47 (35)	0.01
Shortness of breath	5 (3)	14 (11)	0.01
Cough	15 (9)	10 (8)	0.53
Anorexia	32 (19)	26 (20)	0.99
Jaundice	2 (1)	4 (3)	0.21
Lymphadenopathy	0 (0)	0 (0)	NA
Cognitive impairment/mood disturbance	7 (4)	21 (16)	0.0004
Meningitis/encephalitis	0 (0)	5 (4)	0.01
Neutropenia	6 (4)	2 (2)	0.11
Abnormal chest radiograph	11 (7)	2 (2)	0.04

*Values are no. (%) unless indicated otherwise. IQR, interquartile range; NA, not available. †Defined as duration from symptom onset to first seeking medical care. ‡Information regarding a patient’s leukocyte count was available for 68 confirmed and 37 possible cases. §Information regarding a patient’s platelet count was available for 69 confirmed and 36 possible cases. ¶Information regarding a patient’s alanine and aspartate aminotransferase levels was available for 60 confirmed and 36 possible cases.

When compared with persons who had possible illness, a higher proportion who had confirmed illness also had fever (95% vs. 81%; p<0.0001), leukopenia (46% vs. 22%; p = 0.02), and thrombocytopenia (58% vs. 25%; p = 0.001), and a higher proportion of possible cases had recurring fever (35% vs. 22%; p = 0.01), arthralgia (63% vs. 48%; p = 0.02), and cognitive impairment or mood disturbance (16% vs. 4%; p = 0.0004). Approximately one eighth (39/300, 13%) of persons who had HTRF were hospitalized; the percentage hospitalized was similar among persons who had confirmed (20/166, 12%) and possible (19/134, 12%) illness (p = 0.78). There were no deaths.

All laboratory tests performed were either by using PCR or serologic analysis for *B. miyamotoi* infection. PCR was performed for 167 (56%) patients, and serologic analysis was performed for 137 (46%) patients (Table 3). Serologic analysis for paired serum samples was specifically performed for 19 (14%) persons. Among persons who had confirmed illness, 162/164 (99%) had a positive PCR result. Five (3%) of those persons also had a single positive IgM or IgG serologic test result, of whom 2 had a positive IgM result and none had a positive IgG or IgM/IgG combined test result. Among persons who had possible illness, 5/134 (4%) also had PCR performed; all results were negative. No microscopy or culture results were reported for any case.

**Table 3:** Diagnostic results for confirmed and possible cases of hard tick relapsing fever caused by *Borrelia miyamotoi* reported by public health surveillance, United States, 2013–2019*

Characteristic	Confirmed, n = 166†	Possible, n = 134
PCR results		
Detected	162/164 (99)	0/5 (0)
Not detected	0/164 (0)	5/5 (100)
Serologic analysis results		
Single IgM alone		
Positive	2/2 (100)	36/127 (28)
Negative	0/2 (0)	89/127 (70)
Indeterminate	0/2 (0)	2/127 (2)
Single IgG alone		
Positive	0/2 (0)	111/127 (87)
Negative	2/2 (100)	16/127 (13)
Indeterminate	0/2 (0)	0/127 (0)
Single combined IgM/IgG		
Positive	1/2 (50)	6/7 (86)
Negative	0/2 (0)	1/7 (14)
Indeterminate	1/2 (50)	0/7 (0)
Paired serologic samples		
>4-fold change in titer	1/1 (100)	0/18 (0)
<4-fold change in titer	0/1 (0)	18/18 (100)

*Values are no. positive/no. tested (%). †Missing diagnostic information for 2 cases.

Among persons for whom exposure information were available, most (63/69, 91%) reported exposure to ticks or tick habitat; more than half reported a tick bite (46/82, 56%). Travel within (16/70, 23%) or outside (21/79, 27%) the state of residence was infrequently reported. Compared with persons who had possible cases, a higher proportion of persons who had confirmed illness reported exposure to ticks or tick habitat (48/51 [94%] vs. 15/18 [83%]; p = 0.16) or having a known tick bite (37/60, 62% vs. 9/22, 41%, p = 0.09). A total of 15 persons (15/72, 21%) reported receipt of a blood transfusion or organ transplant within the previous 30 days; those occurred among 9 confirmed and 6 possible cases. Among 7 persons who received a blood transfusion and for whom tick exposure information was available, all reported a recent tick bite.

Information on antimicrobial drug treatment was available for 124 (41%) patients. Among those patients, 110 (89%) were given a single antimicrobial drug; 101 (92%) received doxycycline, 5 (5%) amoxicillin, 1 (1%) minocycline, 1 (1%) with cefuroxime, 1 (1%) azithromycin, and 1 (1%) trimethoprim/sulfamethoxazole. Among the 14 patients who received a combination regimen, 13 (93%) were given a regimen of doxycycline and another antimicrobial drug; 1 patient received amoxicillin and cefuroxime.

## Discussion

A total of 300 cases of HTRF caused by *B. miyamotoi* in the United States were identified by using public health surveillance. Case-patients were most commonly male and older adults. Case investigations showed that nonspecific symptoms, including fever and headache, were common, and rash was relatively uncommon. Those clinical features are similar to those of previous large case series of HTRF (*10*,*17*), although the overall proportion of cases with recurring fevers in this report was higher, and recurring fevers were more common among possible cases than among confirmed cases. The percentage of cases associated with hospitalization in this report was lower than reported among a large case series in the northeastern United States (*17*), and there were no reported deaths. However, the data in this summary might include cases reflected in previous case series if those previously reported cases were captured through public health surveillance.

We observed a summertime seasonal pattern for confirmed cases, similar to findings for other infections transmitted by *Ixodes* spp. ticks in the United States (*23*,*35*,*36*). The frequency of HTRF peaked later in the summer than that for Lyme disease (*3*,*23*,*37*). This shifted seasonality supports a role for larval blacklegged ticks in *B. miyamotoi* transmission to humans in the United States because those ticks are more likely to be questing for blood meals during mid-to-late summer than during other tick life stages (*38*), and there is documented transovarial transmission of *B. miyamotoi* (*39*).

Several features differed between confirmed and possible cases. Confirmed cases occurred more commonly among older persons and among male persons than did possible cases. Confirmed cases were more frequently characterized by fever, thrombocytopenia, and increased levels of aminotransferases. Possible cases were more frequently characterized by confusion, mood disorder, abdominal pain, shortness of breath, and recurring fevers. Although possible case-patients tended to have a longer duration of illness before seeking medical care, this difference might simply reflect the bias of the case definition itself, in which direct detection by using PCR was laboratory evidence for the confirmed case definition and a single positive serologic result was laboratory evidence for the possible case definition. Serologic analysis (particularly for IgG) is unlikely to show increased levels during acute illness (*34*). In addition, increased reactivity against GlpQ alone might not be a specific measure of past HTRF infection (*33*,*34*). In this study, all possible case-patients had detectable IgG, suggesting that they might have had illness onset >20 days before testing, and so results might not represent acute *B. miyamotoi* infection or possibly not *B. miyamotoi* infection at all. However, longer duration of illness before seeking medical care for the possible case-patients might enable increased opportunity for observation of recurrent fevers. We observed less striking seasonality of illness onset for possible case-patients than for confirmed case-patients. Those findings collectively decrease our confidence that the possible cases summarized here reflect acute HTRF illness.

The older age distribution among confirmed HTRF cases is similar to that of anaplasmosis and babesiosis cases and differs from Lyme disease cases, even though all are transmitted in the United States by the same species of *Ixodes* ticks (*24*). Lyme disease most commonly affects children 5–14 years of age, as well as older adults (*40*). In contrast, anaplasmosis rarely affects children, and HTRF in children was uncommon in public health surveillance. The older age distribution of anaplasmosis is believed to reflect host susceptibility differences and immune-related factors linked to aging, rather than age-related differences in tick exposure; those potential age-based susceptibility differences might account for the older age distribution associated with persons who have HTRF. However, diverging clinical or diagnostic approaches might be used for children versus adults, such as lower levels of testing or lower clinical awareness that bias the cases identified through public health surveillance toward adults. Because the clinical manifestations of HTRF and anaplasmosis might be similar (*24*), increased clinical education should highlight the potential for anaplasmosis and HTRF to resemble one another.

Fifteen persons in this study reported receiving a blood transfusion or organ transplant in the 30 days before symptom onset. Although no cases of *B. miyamotoi* infection after blood transfusion have been documented, other tickborne pathogens, including *Babesia microti*, *A. phagocytophilum*, and *Ehrlichia chaffeensis*, have caused infections after blood transfusion (*41*,*42*). Spirochetemia might be higher or more prolonged for *B. miyamotoi* infection than for *B. burgdorferi* infection, suggesting that the risk for transmission from blood transfusion is greater for *B. miyamotoi* (*17*,*46*). Nevertheless, all 7 patients who had a confirmed infection and an available exposure history available reported a recent tick bite, suggesting that receipt of blood products or organs might simply reflect risk factors for more severe illness caused by compromised immune status, rather than a potential route of *B. miyamotoi* transmission.

The frequency of recurring febrile episodes in HTRF caused by *B. miyamotoi* is not well understood. The percentage of patients with confirmed illness who had recurring fever (22%) was higher than those reported in a case series from Russia (11%) (*10*) and in a case series from the United States (4%) (*17*). All relapsing fever group borreliae display antigenic variation, a shift in expressed proteins that creates recurring febrile episodes (*43*,*44*). *B. miyamotoi* infection generates a lower level of spirochetemia than its soft tick–transmitted relatives, which might affect severity of illness and ability to generate recurrent fevers in untreated infection (*17*,*45*). Direct detection and treatment early in the illness course could result in clinical cure before antigenic variation occurs. It is difficult to compare the frequency of recurrent fevers in persons who have *B. miyamotoi* infection with persons who have soft tick relapsing fever because of variable methods of ascertainment. Also, no objective capture of details regarding fever recurrence frequency, intervals between febrile episodes, and maximum temperature of each febrile episode were captured through this public health surveillance practice; those details are best captured through detailed clinical case series.

Low clinical awareness, limited availability of PCR testing, and limited specificity of available serologic assays make HTRF case identification challenging. Currently, diagnosis of tickborne infections requires clinicians to order tests specific to each suspected pathogen; the expanded use of multiplex direct detection assays, or tickborne panels, in commercial laboratories might improve detection of *B. miyamotoi* and other tickborne infections. Metagenomics approaches are also an increasing opportunity to improve direct detection of tickborne infections, including co-infections (*46*,*47*). Those approaches are particularly appealing for improved detection of *B. miyamotoi* infection because the spirochetes appear to be present in sufficient quantities in blood for detection by using molecular methods (*17*,*46*,*48*).

Highlighting the usefulness of PCR-based diagnostic methods for *B. miyamotoi* infection, a study found that among patients with PCR-confirmed *B. miyamotoi* infection, the sensitivity of GlpQ IgG was <55% when assayed <20 days after illness onset, which increased to 74%–86% when assayed 21–150 days after illness onset (*33*). GlpQ is a common serologic target for differentiating infection with relapsing fever group borreliae from those that cause Lyme disease. However, there is limited information on its specificity for relapsing fever group borreliae, constraining its clinical usefulness.

Without sufficiently specific serologic assays, the frequency of exposure in the population and characteristics of more mild illness is difficult to ascertain. In addition, GlpQ-based assays cannot distinguish between infection with hard tick and soft tick relapsing fever borreliae, which can co-occur in some areas (i.e., along the Pacific Coast). In those circumstances, a comprehensive exposure history is necessary to direct public health intervention. However, many patients who have suspected tickborne infections, including HTRF, receive doxycycline empirically, which would effectively treat *B. miyamotoi* even if clinicians had not suspected this specific infection. People with mild symptoms who live in a Lyme disease‒endemic area might be more likely to receive empiric therapy.

The first limitation of the surveillance data we describe is that cases identified through passive surveillance probably represent more severe disease because all persons necessarily sought medical care for an illness and obtained laboratory testing. As previously mentioned, persons who have mild symptoms or asymptomatic infections would not be detected by current public health surveillance approaches. Thus, the severity of HTRF caused by *B. miyamotoi* is difficult to reliably measure through this mechanism. Accordingly, the frequency of hospitalization in those data are probably an overestimate caused by inherent ascertainment bias. Second, the nature of public health surveillance activities precludes knowledge of the targets and performance of assays used by commercial laboratories. Third, *B. miyamotoi* might cause human infection in states where the condition is not subject to public health reporting. A recent case of HTRF caused by *B. miyamotoi* was identified in California; that finding, in combination with acarologic and seroprevalence assessments, suggests potential for additional cases of human illness along the Pacific Coast (*28*,*29*,*49*,*50*). Fourth, detailed data on clinical features, such as rash, clinical course and resolution, or immunocompromised status or other medical concurrent conditions, were not collected consistently as part of surveillance-based case investigations, limiting our ability to thoroughly describe the clinical course or examine the effect of immunocompromising conditions or other concurrent conditions on clinical severity or presentation of HTRF caused by *B. miyamotoi* infection. Fifth, data on positive laboratory findings for other tickborne diseases were not regularly compiled as part of public health surveillance; thus, these HTRF cases could reflect patients co-infected with other tickborne diseases.

Public health surveillance in the United States supports that HTRF manifests as a nonspecific febrile illness during the summer months. *B. miyamotoi* is among the group of pathogens transmitted to humans by *Ixodes* spp. ticks, and the clinical manifestations might be similar to that of other tickborne diseases in the same geographic areas. The frequency of asymptomatic or mild illness caused by HTRF that resolves without treatment is not known, nor is the potential for longer-term complications of untreated infection.

At present, *B. miyamotoi* is the only recognized cause of HTRF; if additional pathogens are identified, public health surveillance approaches will necessarily adapt, including through expansion of molecular testing to detect those pathogens. Infections identified through public health surveillance can enable expanded understanding of the clinical spectrum of emerging infectious diseases than what is possible through limited case series or reports, but surveillance depends on clinical suspicion, laboratory diagnostic test access, and state-based public health regulations that enable mandatory reporting of positive laboratory results to the public health system. Ongoing, coordinated public health surveillance for HTRF caused by *B. miyamotoi* will better define its clinical spectrum, severity, incidence, and geographic distribution, and inform associated clinical and public outreach efforts to improve recognition. However, improved access to direct detection of *B. miyamotoi* through unbiased and widely available PCR-based assays, as well as clinically validated serologic markers, are needed to clarify the frequency and severity of the illness.
